# Animal Age-Dependent Susceptibility of Mouse Oocytes to Zearalenone-Induced Developmental Impairment: Roles of Pharmacokinetic Exposure and Intrinsic Oocyte Sensitivity

**DOI:** 10.3390/cells15161444

**Published:** 2026-08-11

**Authors:** Si-Tong Liu, Jun-Gui Zhao, Jia-Li Xu, Min Zhang, Shuai Gong, Hong-Jie Yuan, Jing-He Tan, Ming-Jiu Luo

**Affiliations:** Shandong Provincial Key Laboratory for Livestock Germplasm Innovation & Utilization, College of Animal Science and Technology, Shandong Agricultural University, Tai’an 271018, China; willow9912@163.com (S.-T.L.); 19323107311@163.com (J.-G.Z.); 19845914898@163.com (J.-L.X.); zhangmin@sdau.edu.cn (M.Z.); gongshuai5@sdau.edu.cn (S.G.); yuanhj@sdau.edu.cn (H.-J.Y.); tanjh@sdau.edu.cn (J.-H.T.)

**Keywords:** bioavailability, developmental competence, oocytes, prepuberty, zearalenone

## Abstract

**Highlights:**

**What are the main findings?**

- Oral ZEN exposure impairs oocyte developmental competence, and prepubertal (3-week-old) mice exhibit the greatest susceptibility.
- In vitro, oocytes from prepubertal mice exhibit higher susceptibility to ZEN-induced functional deficits than those from mature adults.

**What is the implication of the main finding?**

- ZEN exposure compromises oocyte developmental competence in a host age-dependent manner.
- The high vulnerability in prepubertal oocytes is associated with both increased ovarian ZEN bioavailability and their intrinsically greater sensitivity to ZEN.

**Abstract:**

**Background:** Zearalenone (ZEN) exposure poses health risks to both humans and animals. Although evidence indicates that ZEN significantly impairs oocyte developmental competence, the underlying mechanisms remain largely unclear. While it is recognized that prepubertal animals are particularly vulnerable to ZEN and that juvenile in vitro embryo transfer represents a promising strategy for accelerating genetic progress, the potential impact of ZEN on developmental competence of prepubertal oocytes remains uninvestigated. **Methods:** In vivo, we administered graded oral doses of ZEN to female mice at prepubertal (3 weeks), peripubertal (6 weeks), and post-pubertal (8 weeks) stages and assessed oocyte developmental potential and related markers and ZEN residues in ovaries and livers following superovulation. In vitro, oocytes isolated from mice of each age group were subjected to graded ZEN concentrations during in vitro maturation, followed by evaluation of oocyte developmental potential. **Results:** This study shows that oral ZEN exposure impairs oocyte developmental competence and cumulus expansion, and disrupts oocyte redox homeostasis, mitochondrial integrity and glutathione biosynthesis in a mouse age-specific manner, with prepubertal (3-week-old) mice exhibiting the greatest susceptibility. Assessment of systemic ZEN disposition shows that ZEN concentrations in both ovarian and hepatic tissues were significantly higher in 3-week-old mice than in 6- and 8-week-old mice. Furthermore, exposure of oocytes to graded ZEN concentrations during in vitro maturation demonstrate that oocytes from prepubertal mice exhibit intrinsically heightened susceptibility to ZEN-induced functional deficits relative to those from sexually mature adults. **Conclusions:** This study demonstrates that ZEN exposure compromises oocyte developmental competence in a host age-dependent manner, and the heightened vulnerability in prepubertal oocytes is associated with both increased ovarian ZEN bioavailability and their intrinsically greater sensitivity to ZEN.

## 1. Introduction

Zearalenone (ZEN) is frequently detected in cereal grains used for human food and animal feed, with a reported detection rate of approximately 46% and a maximum concentration reaching 3.05 mg kg^−1^ [1]. Consequently, ZEN can easily enter the food/feed supply chain, posing health risks to both humans and animals. Accumulating evidence indicates that ZEN exerts significant toxic effects on oocyte development and maturation. For example, in vitro exposure of porcine oocytes to ZEN reduces their maturation and developmental competence and induces abnormalities in spindle assembly and blastomere ploidy [2]. Similarly, feeding mice with ZEN-contaminated diets significantly impairs oocyte quality, leading to aberrant spindle formation, disrupted cortical granule distribution, and mitochondrial dysfunction [3]. Moreover, in vivo studies in mice demonstrate that ZEN exposure dose-dependently compromises oocyte competence by inducing oxidative stress and disrupting chromatin architecture and gene transcription [4]. Despite these findings, the underlying mechanisms through which ZEN impairs oocyte development and maturation remain largely unclear.

Prepubertal animals are widely recognized as a particularly vulnerable subpopulation to mycotoxin exposure, primarily due to ontogenetic immaturity of key metabolic and excretory organs including the liver, kidneys, and gastrointestinal tract, which results in altered toxicokinetics and higher systemic bioavailability of xenobiotics [5]. Additionally, their relatively greater food/feed intake per unit body weight may further elevate dietary exposure to foodborne contaminants such as ZEN [6]. Indeed, ZEN has been epidemiologically linked to numerous outbreaks of hyperestrogenism in livestock, with prepubertal gilts consistently identified as the most sensitive cohort in swine [7]. Catteuw et al. [8] conducted comparative toxicokinetic studies of ZEN and its major phase II metabolites, including zearalenone-14-glucoside (ZEN14G) and zearalenone-14-sulfate (ZEN14S), in 4- and 8-week-old pigs. Their findings revealed pronounced age-dependent differences attributable to developmental variations in intestinal permeability, adipose tissue distribution, gastrointestinal transit time, and biotransformation capacity; notably, the absorbed fraction of ZEN14G was significantly higher in younger animals (94% vs. 61% in 4- vs. 8-week-olds, respectively).

From a production standpoint, earlier onset of puberty in breeding females is generally advantageous, as it shortens the non-productive rearing period and reduces cumulative feed expenditures [9]. For example, advancing puberty by one to two weeks in gilts can yield measurable economic benefits. Green et al. [10] demonstrated that prepubertal ZEN exposure suppressed mean serum luteinizing hormone (LH) concentrations, though this effect did not translate into delayed puberty onset when ZEN was withdrawn from the diet at least two weeks prior to boar exposure. While juvenile in vitro embryo transfer (JIVET) represents a promising strategy for accelerating genetic progress by enabling blastocyst production from prepubertal oocytes and thereby reducing generation intervals [11], the potential impact of ZEN on the developmental competence and molecular integrity of prepubertal oocytes remains uninvestigated. Consequently, a rigorous, mechanism-informed assessment of the heightened susceptibility of prepubertal animals to naturally occurring mycotoxins, particularly ZEN, is both scientifically warranted and critical for evidence-based risk management in animal agriculture.

The present study aimed to determine whether ZEN exposure compromises oocyte developmental competence in a host age-dependent manner, and whether the heightened vulnerability in prepubertal mice arises from increased systemic or ovarian ZEN bioavailability or intrinsically greater sensitivity of prepubertal oocytes to ZEN. To address these questions, we administered graded oral doses of ZEN to female mice at three distinct developmental stages—prepubertal (3 weeks), peripubertal (6 weeks), and post-pubertal (8 weeks)—and assessed oocyte developmental potential and related markers and ZEN residues in ovaries and livers via liquid chromatography–tandem mass spectrometry (LC–MS/MS), following superovulation. Complementary in vitro experiments were conducted by subjecting oocytes isolated from mice of each age group to defined ZEN concentrations during in vitro maturation (IVM), followed by evaluation of oocyte developmental potential.

## 2. Materials and Methods

We carried out the study according to the relevant guidelines and regulations. We conducted mouse care and handling strictly in accordance with the guidelines issued by the Animal Care and Use Committee of Shandong Agricultural University, China (Permit number: SDAUA-2019-004). We purchased all the chemicals and reagents used in this study from Sigma-Aldrich Corp. (St. Louis, MO, USA), unless specified otherwise.

### 2.1. Intragastric Administration and Superovulation in Mice

Mice of the Kunming strain, originally derived from ICR (CD-1) mice, were used in this study. Healthy female mice with consistent body weight at 3, 6, and 8 weeks of age were randomly divided into a ZEN-treated group and a control group. ZEN was dissolved in DMSO to prepare a 3.33 mg/mL stock solution, which was stored in the dark and diluted with physiological saline before administration. The treated group received ZEN solution by gavage, while the control group was gavaged with the same volume of DMSO–saline mixture. All mice were gavaged daily between 16:00 and 17:00 for 8 days. At 24 h after the last gavage, mice were injected with 10 IU eCG, and at 48 h after eCG injection, mice were euthanized to obtain oocytes. Liu et al. [12] found that treatment of pregnant mice with 40 µg/kg BW of ZEN impaired fetal germ cell meiotic progression and primordial follicle assembly. Pan et al. [4] reported that treatment of adult mice with 500 µg/kg BW of ZEN significantly damaged their oocytes. Therefore, we tested 50, 100, 150 μg/kg BW of ZEN on oocyte developmental potential to determine the no observed effect level (NOEL) of ZEN on mouse oocytes.

### 2.2. Oocyte Recovery, In Vitro Maturation (IVM), Activation, and Embryo Culture

Ovaries were collected following euthanization of mice, and the large follicles on the ovary were ruptured in M2 medium using a needle to recover cumulus–oocyte complexes (COCs). Under a stereomicroscope, COCs with more than three layers of unexpanded cumulus cells and an oocyte larger than 70 µm in diameter with homogeneous cytoplasm were selected for experiments.

The selected COCs were cultured in 100 µL micro drops of maturation medium under mineral oil, with approximately 25–30 COCs per drop. The maturation medium was TCM-199 (Gibco, Grand Island, NY, USA) supplemented with 10% *v*/*v* fetal bovine serum (Gibco, Grand Island, NY, USA), 1 µg/mL 17β-estradiol, 24.2 mg/mL sodium pyruvate, 0.05 IU/mL follicle stimulating hormone (FSH), 0.05 IU/mL luteinizing hormone (LH), and 10 ng/mL epidermal growth factor (EGF). The medium was pre-equilibrated for 3 h at 37 °C in a humidified atmosphere of 5% CO_2_ before use.

At 24 h of maturation culture, mouse COCs were pipetted in M2 medium containing 0.1% hyaluronidase to remove cumulus cells. The cumulus-denuded oocytes (DOs) with a first polar body were washed twice in M2 medium and once in activation medium. They were then incubated in activation medium for 6 h at 37 °C in a humidified atmosphere with 5% CO_2_. The activation medium was Ca^2+^-free CZB supplemented with 10 mM SrCl_2_ and 5 µg/mL cytochalasin B (CB) at a 1:100:900 ratio and pre-equilibrated for 3 h before use. After activation treatment, oocytes were examined under a phase-contrast microscope, and those with one or two well-developed pronuclei were considered activated. Activated oocytes were cultured in regular CZB medium (25–30 oocytes per 100 µL drop) at 37 °C with 5% CO_2_ in air, and 5.5 mM glucose was added to the CZB medium when embryos developed beyond the 3- or 4-cell stage. Embryos were examined at 24, 48, and 96 h post-activation to record the number of 2-cell, 4-cell, and blastocyst embryos, respectively.

### 2.3. One-Step Real-Time PCR Analysis of mRNA Expression

The DOs were lysed to extract RNA using a CellAmp Direct Prep Kit (3733; TakaraBio, Dalian, China). The lysate was stored at −80 °C until use. mRNA expression in oocytes was quantified using the One Step TB Green^®^ PrimeScript™ PLUS RT-PCR Kit (RR096A; TakaraBio, Dalian, China). The total volume of the amplification reaction was 10 µL. The reaction solution consisted of 1 µL of total RNA, 5 µL of 2 × One Step TB Green RT-PCR Buffer, 0.6 µL of TaKaRa Ex Taq HS Mix, 0.2 µL of PrimeScript PLUS RTase Mix, 2.4 µL of DEPEC-ddH_2_O, and 0.4 µL (10 µM) each of the forward and reverse gene-specific primers. The reaction was carried out in an Mx3005P (Stratagene, Valencia, CA, USA) as follows: (1) reverse transcription for one cycle at 42 °C for 5 min followed by 95 °C for 10 s; (2) amplification for 40 cycles at 95 °C for 5 s followed by 34 s at the annealing temperature (53 °C for Mater; 5 °C for Prkce; 58 °C for Bax, Bcl2 and Zar1); (3) final dissociation reaction at 95 °C for 15 s, 60 °C for 1 min and 95 °C for 15 s. H2afz was used as the internal reference gene to calculate relative expression with the 2^−△△CT^ method. The gene-specific primers used are listed in Table 1.

### 2.4. Measurement of Cumulus Expansion Area

Collected COCs were cultured in 100 µL drops at a density of 25–30 COCs per drop and incubated for 18 h for IVM at 37.5 °C in a humidified atmosphere of 5% CO_2_. The COCs were photographed under an inverted microscope before maturation (BM) and after maturation (AM) culture at the same magnification and imaging parameters. The cumulus expansion areas of COCs at BM and AM were measured, and the ratio of AM/BM was calculated using Image Pro Plus 6.0 software (Media Cybernetics, Rockville, MD, USA).

### 2.5. Measurement of Intra-Oocyte Reactive Oxygen Species (ROS)

Intra-oocyte ROS were measured by detecting H_2_O_2_ concentrations using 2′,7′-dichlorodihydro-fluorescein diacetate (DCHF-DA). A stock solution (1 mM) of DCFH-DA was prepared in DMSO and stored in the dark at −20 °C. Immediately before use, the stock solution was diluted to 10 µM in M2 medium. Cumulus cells were removed from oocytes that had matured to the MII stage after 16 h of maturation culture, and the DOs were stained in the working solution at 37 °C for 10 min. After being washed several times in M2 medium, the oocytes were placed on a slide, covered with a coverslip, and observed under a Leica fluorescence microscope (Leica Microsystems, Wetzlar, Germany). Fluorescence signals were detected at an excitation wavelength of 488 nm. All images were captured using fixed microscope parameters to ensure data consistency. Fluorescence intensity of individual oocytes was analyzed using Image-Pro Plus 6.0 software.

### 2.6. Measurement of Mitochondrial Membrane Potential

Mitochondrial membrane potential (MMP) was measured in MII oocytes matured in vitro for 16 h using a JC-1 MMP detection kit (C2006; Beyotime Biotechnology, Shanghai, China). DOs were washed three times in M2 medium and incubated in working solution (M2 medium and JC-1 staining solution mixed at 1:1) at 37 °C for 25 min. After incubation, oocytes were washed three times with JC-1 staining buffer and observed under a Leica laser scanning confocal microscope (Leica Microsystems, Wetzlar, Germany). JC-1 aggregates (red fluorescence) were detected at 590 nm using the TRITC channel, while JC-1 monomers (green fluorescence) were detected at 512 nm using the FITC channel. The same oocyte was observed under both channels at the same magnification, and images from control and treatment groups were captured with identical parameters. Fluorescence intensity was analyzed using Image-Pro Plus software, and the ratio of red to green fluorescence was calculated to evaluate mitochondrial depolarization.

### 2.7. Determination of Glutathione Content in Oocytes

Total glutathione (GSX) and oxidized glutathione (GSSG) contents in oocytes were measured using a GSH and GSSG Assay Kit (S0053; Beyotime Biotechnology, Shanghai, China). MII oocytes matured in vitro for 16 h were used for glutathione detection. Forty cultured COCs were collected, and cumulus cells were removed with hyaluronidase. The DOs were washed three times in M2 medium and collected into a 1.5 mL centrifuge tube. After centrifugation at 3000× *g* for 5 min, the supernatant was discarded. Then, 30 µL of freshly prepared protein removal reagent was added and mixed thoroughly. The samples were frozen at −80 °C and thawed at room temperature repeatedly for at least three cycles. After standing at 4 °C for 5 min, the samples were centrifuged at 10,000× *g* for 10 min. The supernatant was collected for immediate analysis or stored at −80 °C until analysis. GSX and GSSG levels were determined according to the manufacturer’s instructions. Standard curves were prepared using GSX or GSSG standards at concentrations of 0.5, 1, 2, 5, 10 and 15 µM, and absorbance was measured at 405 nm with a microplate reader (Infinite F50; Tecan Group Ltd., Männedorf, Switzerland). The reduced glutathione (GSH) content was calculated by subtracting GSSG from GSX. The concentrations of GSSG and GSH (pmol per oocyte) and the GSH/GSSG ratio were calculated based on the oocyte number.

### 2.8. Measurement of ZEN in Ovaries and Livers of Female Mice at Different Ages

ZEN content in tissue homogenate samples was determined by LC–MS/MS, referring to GB 5009.209-2016. Mice ovaries and livers were collected at 46–48 h after eCG injection. Ovarian and liver tissues were rinsed with normal saline, homogenized and stored at −80 °C until use.

A sample was weighed, mixed with sodium chloride and 5 mL of extraction solution by vortexing, then ultrasonically extracted for 30 min. After centrifugation at 4 °C for 15 min, the supernatant was collected and mixed with 2 mL PBS buffer.

Purification was performed using an immunoaffinity column: activated with methanol and equilibrated with PBS, the sample was loaded at 0.5 mL/min. After rinsing with PBS to remove impurities, ZEN was eluted with 1 mL of methanol, and the eluent was filtered through a 0.22 µm membrane before LC–MS/MS detection.

The external standard method was used to draw the standard curve (R^2^ ≥ 0.999) for calculating ZEN concentration from sample peak areas. Each sample had 3 parallel replicates, and mean value and RSD were calculated to ensure result accuracy and repeatability. In the calibration curve for ZEN (Figure S1), calibration standards were in the range of 1.0–50.0 ng/mL, with a linear regression fit of R^2^ = 0.9993. The back-calculated accuracy of all calibration standards ranged from 91.9% to 108.1%, meeting the acceptance criteria of ±15% for bioanalytical method validation. In the residual plot for the ZEN calibration curve (Figure S2), all relative residuals fell strictly within the ±15% acceptance range (distribution from −8.1% to + 8.1%), with no obvious systematic bias, indicating that the linear regression model provided a satisfactory fit. In the MRM chromatograms of ZEN in tissue homogenates (Figure S3), the background signal at the ZEN retention time (3.31 min) was 4.14 × 10^3^ cps, corresponding to only 1.2% of the peak area of the 1 ng/mL standard, confirming the absence of significant endogenous matrix interference. Matrix-derived components eluting at 2.6 min and 3.8–5.5 min were well separated from the ZEN peak, further ensuring method specificity. Liver homogenate spiked with ZEN at 10 ng/mL, RT = 3.31 min, peak area = 3.998 × 10^6^ cps, with a spike recovery of 105%, falling within the acceptable range (85–115%).

The matrix-matched calibration curve exhibited good linearity within the range of 1.00–50.00 ng/mL (R^2^ = 0.9993). The limit of detection (LOD) and limit of quantification (LOQ) were determined to be 4 μg/kg and 10 μg/kg in tissue matrices, respectively. The overall method accuracy was assessed via a matrix spiking experiment; a tissue sample fortified with ZEN standard at 10 ng/mL yielded a recovery rate of 105%. The assay was conducted on an AB SCIEX QTRAP 5500 system using the MRM transition of *m*/*z* 317.0 → 175.0. Sample preparation strictly followed the immunoaffinity column cleanup proce-dure as specified in GB 5009.209-2016. The IAC-based sample preparation provides high selectivity for ZEN, effectively reducing matrix-induced ion suppression, which is con-sistent with previous reports using similar IAC-LC–MS/MS approaches for ZEN determination in biological matrices.

### 2.9. Statistical Analysis

All data were analyzed using the Statistics Package for Social Science (SPSS 20.0, IBM Corp., Armonk, NY, USA). Percentage data were subjected to arcsine transformation before analysis. One-way analysis of variance (ANOVA) was used when more than two groups were compared, followed by Duncan’s multiple range test to determine significance of differences. Independent-samples *t*-test was used for comparisons between two groups. All data are presented as means ± SEM. Differences were considered statistically significant at *p* < 0.05. Each experiment was repeated at least three times unless otherwise stated.

To explore the interaction between ZEN exposure and mouse age on oocyte developmental competence, a 2 × 3 (age × dose) completely randomized factorial design was employed for the in vitro ZEN exposure experiment. Factor A (age) included three age levels—3 wk, 6 wk, and 8 wk. Factor B (ZEN dose) included three exposure levels—0, 5, and 7 µM. Full factorial combination yielded nine treatment groups, with *n* = 5 per group (some *n* = 6), totaling 48 observations. Measured outcomes included five developmental rates: rates of maturation, activation, 2C, 4C, and Blasts. Two-way ANOVA with interaction was used. Model: Y = μ + α_i_(age) + β_j_(dose) + (αβ)_ij_(interaction) + ε. Type II sum of squares decomposition (suitable for balanced/unbalanced designs) was applied to test three hypotheses: main effect of age, main effect of dose, and interaction effect. Post hoc multiple comparisons were conducted using Tukey’s HSD (Honestly Significant Difference) test to compare all pairs among the nine treatments. Significance level: α = 0.05; significance markers: * *p* < 0.05, ** *p* < 0.01, *** *p* < 0.001, ns = not significant.

## 3. Results

### 3.1. Effects of ZEN Gavage on Oocyte Maturation and Developmental Competence in Female Mice Across Different Developmental Ages

Female mice at ages of 3 weeks, 6 weeks, and 8 weeks were orally administered ZEN at varying doses (0, 50, 100, or 150 µg/kg body weight) daily for eight consecutive days. Age-matched control animals received an equivalent volume of vehicle (DMSO). Twenty-four hours after the final administration, all mice underwent superovulation with eCG; 46–48 h later, they were humanely euthanized, and the germinal vesicle (GV) oocytes were retrieved for in vitro maturation (IVM). Following IVM, MII-arrested oocytes were either subjected to strontium chloride (Sr^2+^)-induced parthenogenetic activation at 24 h of IVM to assess embryonic developmental progression or processed at 18 h of IVM for RT-qPCR analysis of gene expression.

**Figure 1:**
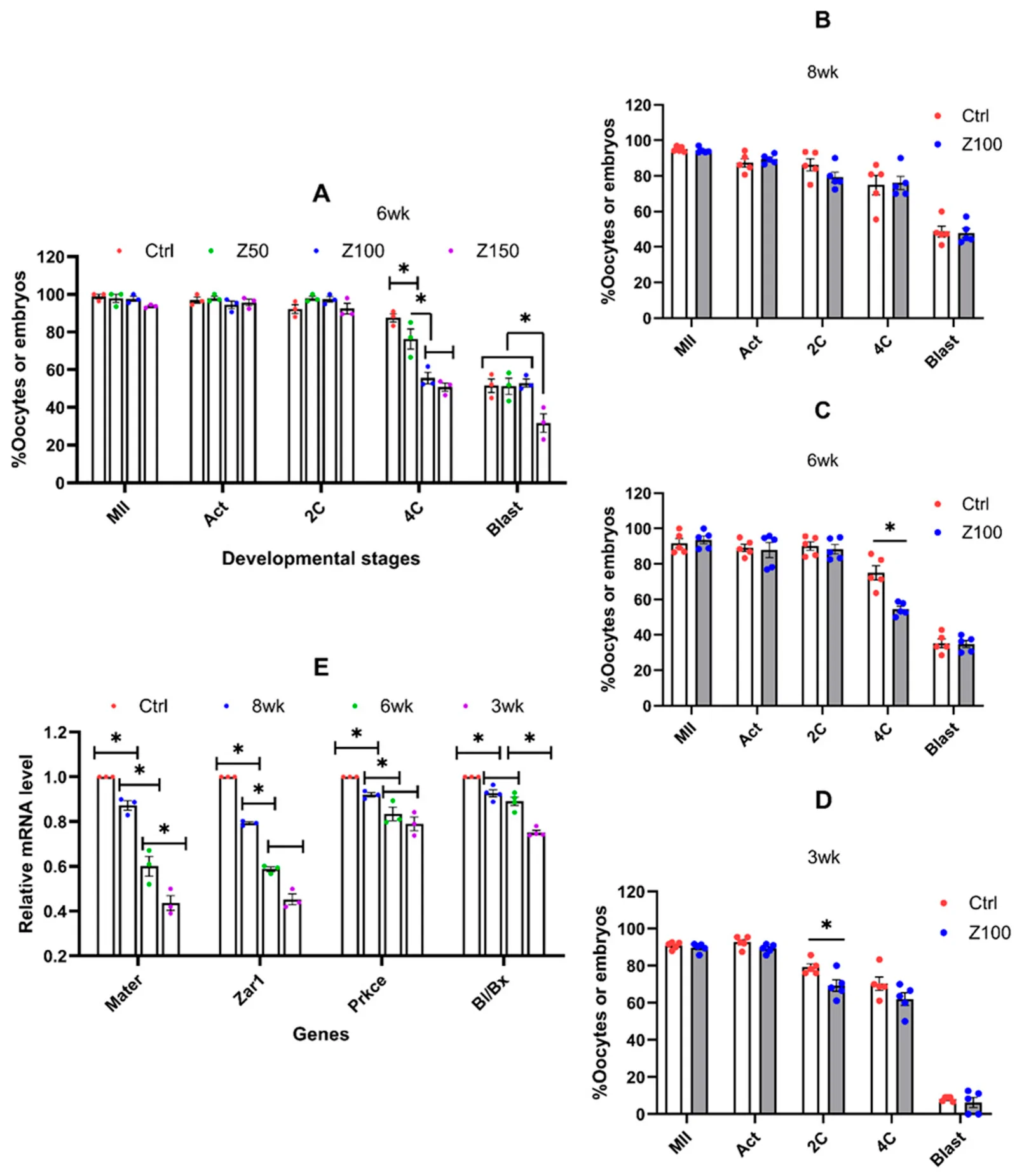
Effects of ZEN gavage on maturation and developmental potential of oocytes from mice at different ages. Panels (**A**–**D**) show percentages of MII and Act oocytes, and 2C, 4C or Blast embryos following in vitro maturation of oocytes from Ctrl or ZEN-treated 6-, 8-, 6- and 3-week-old mice, respectively. Each treatment was repeated 5 times, with each replicate containing about 25–30 oocytes from 2 mice. Percentages of MII and Act oocytes, and 2C, 4C and Blast embryos were calculated from cultured, MII, and Act oocytes, and 2C and 4C embryos, respectively. Panel (**E**) shows relative mRNA levels of Mater, Zar1, Prkce genes and ratios of Bcl2/Bax (Bl/Bx) genes in MII oocytes from different mouse groups. Each treatment was repeated 3 times, with each replicate including 30 oocytes from 2 mice. The value in Ctrl mice was set to one, and values in other groups were expressed relative to one. (*) Indicates significant difference (*p* < 0.05) between means. For dot plots, each individual dot represents the measured value from one independent biological replicate, and horizontal lines within groups indicate the group mean value.

In 6-week-old mice treated with 50, 100, or 150 µg/kg ZEN, no significant differences were observed in the proportions of MII oocytes, activated (Act) oocytes, or 2-cell (2C) embryos across treatment groups. However, the proportion of 4-cell (4C)/2C embryos was significantly reduced in the 100 and 150 µg/kg groups relative to both the vehicle control and the 50 µg/kg group (Figure 1A). Furthermore, blastocyst (Blast) formation rates were significantly lower only in the 150 µg/kg group compared with all other groups.

When a fixed dose of 100 µg/kg ZEN was administered to mice aged 3, 6, and 8 weeks, age-dependent effects on embryonic development became evident: no significant alterations were detected in oocytes from 8-week-old mice (Figure 1B); in contrast, the 4C/2C embryo rate was significantly diminished in 6-week-old mice (Figure 1C), while the rate of 2C embryo/Act oocytes was significantly reduced in 3-week-old mice (Figure 1D). Notably, when the 4C embryo rate was calculated relative to the number of Act oocytes (rather than 2C embryos), the difference between control and ZEN-treated 3-week-old mice reached statistical significance (56.0 ± 4.1% vs. 42.7 ± 1.7%, *p* < 0.05).

Consistent with these functional deficits, RT-qPCR analysis revealed that ZEN exposure (100 µg/kg) significantly downregulated mRNA expression of key competence-beneficial genes including Mater, Zar1, and Prkce as well as the anti-apoptotic Bcl2/Bax transcript ratio in oocytes isolated from all three age groups. The magnitude of suppression was most pronounced in oocytes from 3-week-old mice (Figure 1E).

Collectively, these findings demonstrate that oral ZEN exposure impairs oocyte developmental competence in a mouse developmental stage-specific manner, with prepubertal (3-week-old) mice exhibiting the greatest susceptibility.

### 3.2. Effects of ZEN Gavage on Cumulus Expansion in Oocytes from Female Mice at Distinct Developmental Stages

Female mice aged 3, 6, and 8 weeks were orally administered ZEN at a dose of 100 µg/kg body weight. Forty-eight hours after eCG priming, mice were euthanized to collect GV-stage oocytes. These oocytes were subjected to IVM for 18 h before cumulus expansion assessment.

**Figure 2:**
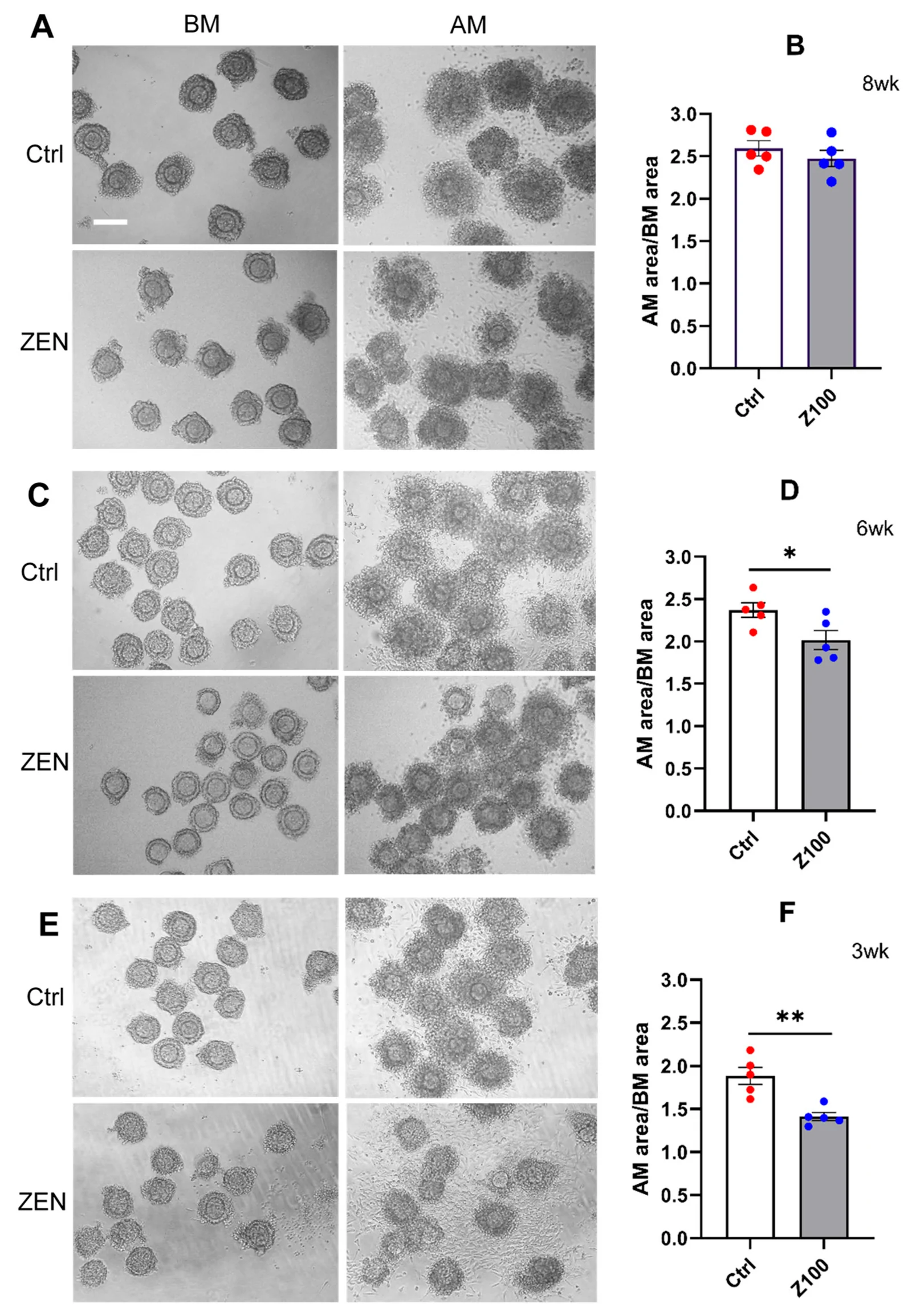
Effects of ZEN gavage on cumulus expansion of oocytes from mice at different ages. Panels (**A**,**C**,**E**) include micrographs showing COCs from Ctrl or ZEN (Z100)-treated 8-, 6- and 3-week-old mice, respectively, before (BM) and after maturation (AM) culture. Bar = 100 µm. Panels (**B**,**D**,**F**) show AM/BM area of cumulus in oocytes from Ctrl or Z100-treated 8-, 6- and 3-week-old mice, respectively. Each treatment was repeated 5 times, with each replicate including 20–25 oocytes from 2 mice. (* and **) Indicate significant differences at *p* < 0.05 and 0.005, respectively, between means. For dot plots, each individual dot represents the measured value from one independent biological replicate, and horizontal lines within groups indicate the group mean value.

Cumulus expansion was quantified as the ratio of AM to BM cumulus area. While no significant difference in AM/BM ratio was observed between vehicle- and ZEN-treated 8-week-old mice (Figure 2A,B), ZEN exposure significantly reduced this ratio in 6-week-old (Figure 2C,D) and, more markedly, in 3-week-old mice (Figure 2E,F) relative to their respective controls. Statistical analysis revealed progressively higher *p*-values across age groups (3 < 6 < 8 weeks). The results suggest an age-dependent attenuation of ZEN-induced impairment in cumulus expansion—with prepubertal mice exhibiting the greatest sensitivity. Given that robust cumulus expansion is strongly correlated with oocyte developmental competence, these findings demonstrate that oral ZEN exposure compromises oocyte quality in an animal developmental stage-specific manner, with maximal vulnerability during the prepubertal period.

### 3.3. Effects of ZEN Gavage on Intra-Oocyte Redox Status in Oocytes from Female Mice at Distinct Developmental Stages

Female mice aged 3, 6, and 8 weeks were orally administered ZEN at a dose of 100 µg/kg body weight. Forty-eight hours after eCG priming, mice were euthanized to collect GV-stage oocytes. These oocytes were subjected to IVM for 16 h before reactive oxygen species (ROS) and mitochondrial membrane potential (MMP) analyses.

**Figure 3:**
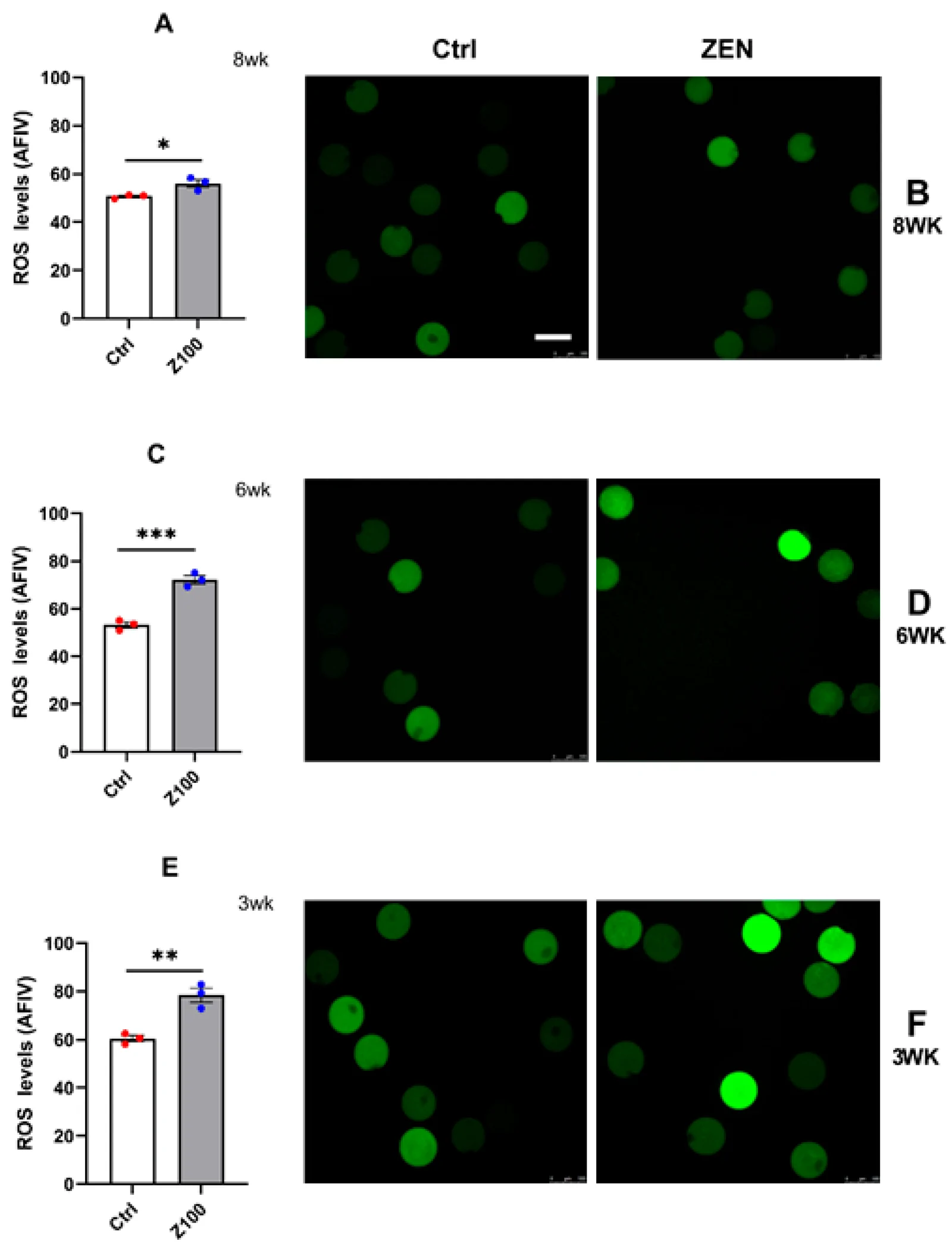
Effects of ZEN gavage on levels of intra-oocyte ROS. Panels (**A**,**B**), (**C**,**D**) and (**E**,**F**) show ROS levels (average fluorescence intensity value, AFIV) in MII oocytes from Ctrl or Z100-treated 8-, 6- and 3-week-old mice, respectively. Each treatment was repeated 3 times, with each replicate containing 25–30 oocytes from 2 mice. (*, **, and ***) Indicate significant differences at *p* < 0.05, 0.005 and 0.001, respectively, between means. For dot plots, each individual dot represents the measured value from one independent biological replicate, and horizontal lines within groups indicate the group mean value. Panels (**B**,**D**,**F**) contain confocal images showing FIV of ROS (H_2_O_2_) in Ctrl or oocytes from Z100-treated mice at the ages of 8, 6 and 3 weeks, respectively. Scale bar = 80 μm.

**Figure 4:**
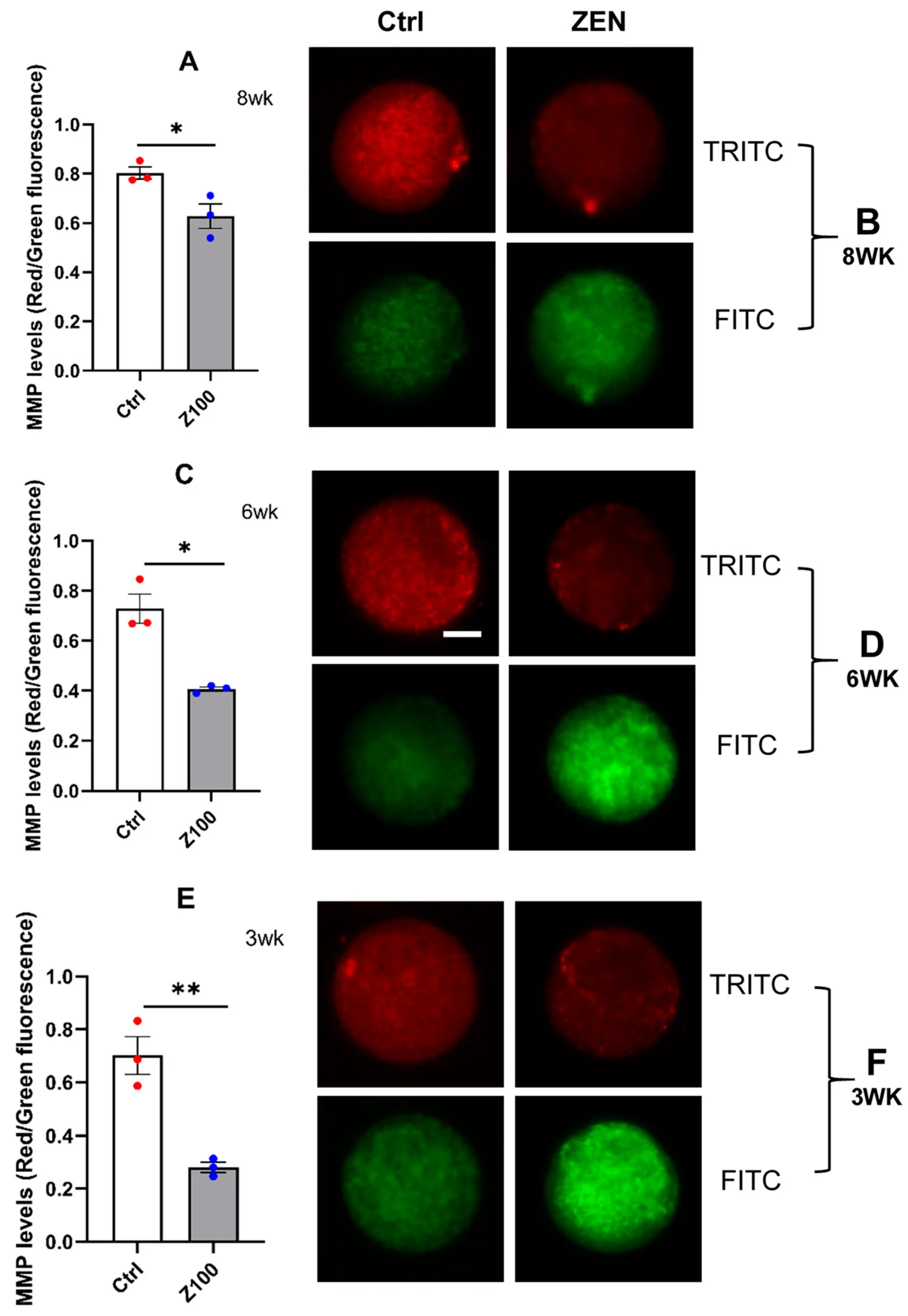
Effects of ZEN gavage on levels of mitochondrial membrane potential (MMP). Panels (**A**,**B**), (**C**,**D**) and (**E**,**F**) show MMP levels (Red/Green fluorescence) in MII oocytes from Ctrl or Z100-treated 8-, 6- and 3-week-old mice, respectively. Each treatment was repeated 3 times, with each replicate containing 25–30 oocytes from 2 mice. (* and **) Indicate significant differences at *p* < 0.05 and 0.005, respectively, between means. For dot plots, each individual dot represents the measured value from one independent biological replicate, and horizontal lines within groups indicate the group mean value. Panels (**B**,**D**,**F**) contain confocal images showing MMP in oocytes from Ctrl or Z100-treated mice at the ages of 8, 6 and 3 weeks, respectively. Scale bar = 23 μm.

Treatment with ZEN significantly increased intra-oocyte ROS levels (Figure 3) and significantly decreased MMP (Figure 4) across all three age groups. This reciprocal dysregulation (elevated oxidative stress coupled with mitochondrial depolarization) indicates that in vivo ZEN exposure induces mitochondrial dysfunction and consequent oxidative damage in oocytes. Importantly, the magnitude of these effects diminished with increasing age: both ROS elevation and MMP reduction were strongest in 3-week-old mice. Collectively, these results establish that ZEN disrupts oocyte redox homeostasis and mitochondrial integrity in an animal age-dependent fashion, with prepubertal oocytes displaying heightened susceptibility to ZEN-mediated oxidative insult.

### 3.4. Effects of ZEN Gavage on Intra-Oocyte Glutathione Homeostasis in Oocytes Derived from Female Mice of Different Ages

**Figure 5:**
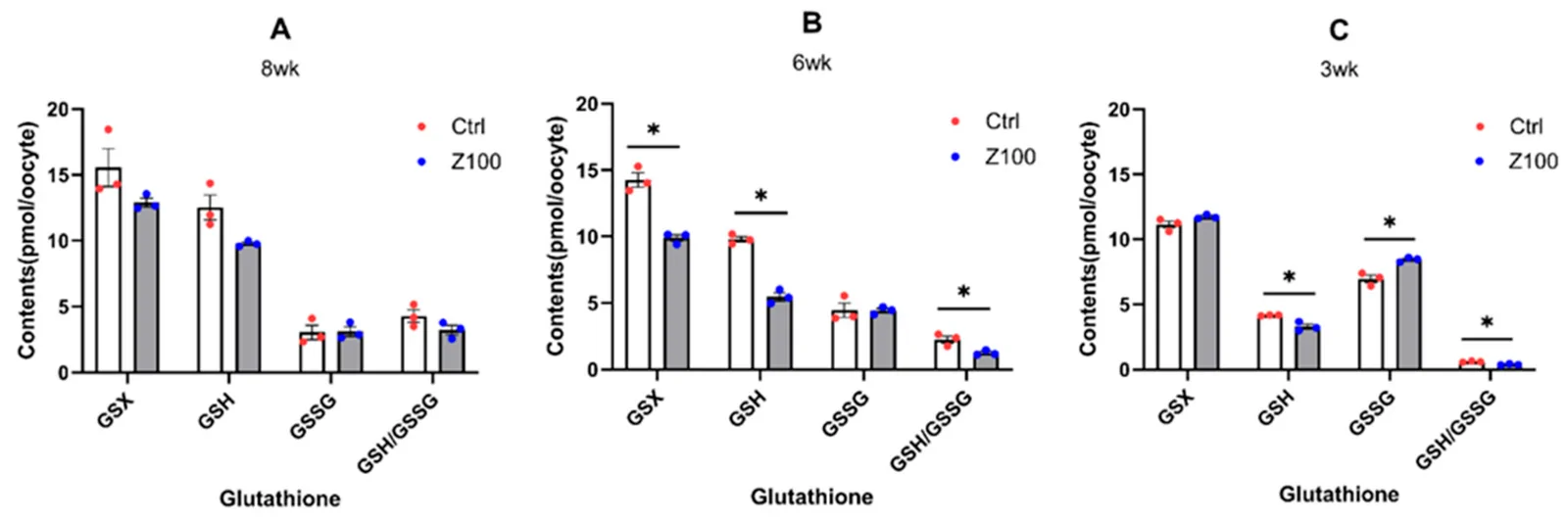
Effects of ZEN gavage on intraoocyte contents of glutathione. Panels (**A**–**C**) show levels of GSX, GSH, GSSG and GSH/GSSG in MII oocytes from Ctrl or Z100-treated 8-, 6- and 3-week-old mice, respectively. Each treatment was repeated 3 times, with each replicate including 40 oocytes from 2 mice. (*) Indicates significant difference (*p* < 0.05) between means. For dot plots, each individual dot represents the measured value from one independent biological replicate, and horizontal lines within groups indicate the group mean value.

Female mice aged 3, 6, and 8 weeks were orally administered ZEN at a dose of 100 µg/kg body weight. Forty-eight hours after eCG administration, mice were euthanized to collect GV-stage oocytes. These oocytes were subsequently subjected to IVM for 16 h to obtain MII-stage oocytes, which were then analyzed for GSX, GSH, GSSG, and the GSH/GSSG ratio. ZEN exposure did not significantly alter any of these glutathione parameters in oocytes from 8-week-old mice (Figure 5A). In contrast, oocytes from 6-week-old mice exhibited significant reductions in GSX, GSH concentration, and the GSH/GSSG ratio following ZEN treatment (Figure 5B). In oocytes from 3-week-old mice, ZEN exposure significantly decreased GSH levels while concurrently increasing GSSG levels, resulting in a marked reduction in the GSH/GSSG ratio (Figure 5C). Notably, baseline GSX levels in untreated control oocytes declined progressively with decreasing age: GSX content in 3-week-old mice was markedly lower than that in both 6- and 8-week-old mice. Collectively, these findings indicate that in vivo ZEN exposure induces age-dependent oxidative stress in murine oocytes, primarily through disruption of glutathione biosynthesis and redox balance.

### 3.5. Effects of ZEN Gavage on Tissue Distribution and Residual Concentrations of ZEN in Female Mice of Different Ages

To assess systemic ZEN disposition, ovaries and livers were harvested 46–48 h after eCG administration (concurrently with oocyte recovery). Tissue homogenates were analyzed via LC–MS/MS for quantification of ZEN residues. Results revealed an inverse correlation between mouse age and ZEN accumulation: ZEN concentrations in both ovarian and hepatic tissues were significantly higher in 3-week-old mice compared to those in 6- and 8-week-old mice (Table 2). This suggests that increased ovarian bioavailability of ZEN in prepubertal mice compromises oocyte developmental competence.

### 3.6. Effects of In Vitro ZEN Exposure on Oocyte Maturation, Embryonic Developmental Potential, and Cumulus Cell Expansion Across Age Groups

**Figure 6:**
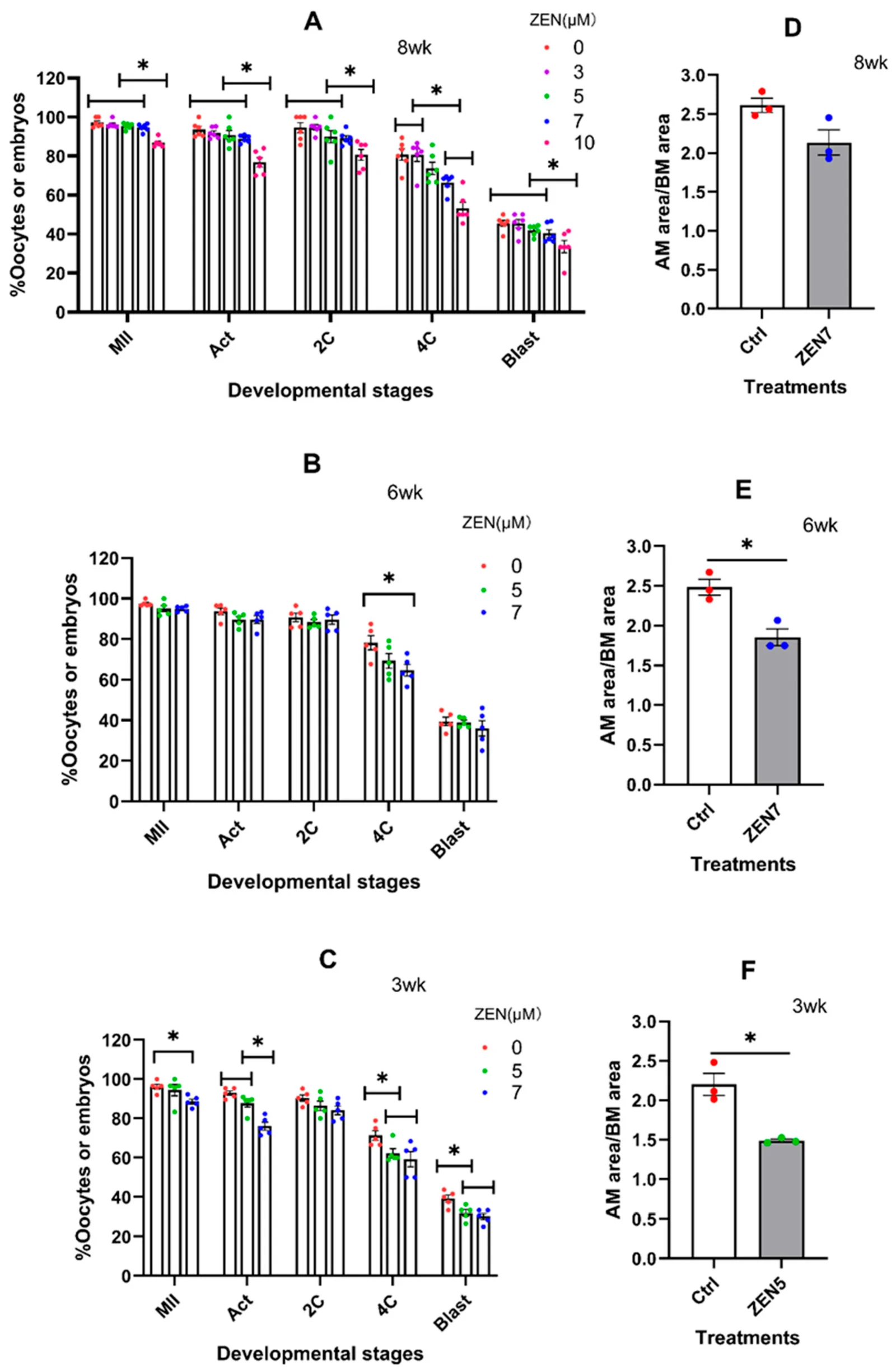
Effects of in vitro ZEN exposure on maturation/developmental potential and cumulus expansion of oocytes from mice of different ages. Panels (**A**–**C**) show percentages of MII or Act oocytes, 2C, 4C or Blast embryos after oocytes from 8-, 6- and 3-week-old mice, respectively, matured in the presence of different concentrations of ZEN. Percentages of MII and Act oocytes, and 2C, 4C and Blast embryos were calculated from cultured, MII, and Act oocytes, and 2C and 4C embryos, respectively. Each treatment was repeated 5 times, with each replicate containing about 25–30 oocytes from 2 mice. Panels (**D**–**F**) show AM/BM area of cumulus expansion after oocytes from 8-, 6- and 3-week-old mice, respectively, matured without (Ctrl) or with 7 µM (8- or 6-week-old mice) or 5 µM (3-week-old mice) ZEN. Each treatment was repeated 5 times, with each replicate including 20–25 oocytes from 2 mice. (*) Indicates significant difference (*p* < 0.05) between means. For dot plots, each individual dot represents the measured value from one independent biological replicate, and horizontal lines within groups indicate the group mean value.

Oocytes isolated from 3-, 6-, and 8-week-old mice were subjected to IVM in the presence of graded concentrations of ZEN (0–10 µM). Embryo development was assessed following parthenogenetic activation after 24 h of IVM, whereas cumulus expansion was evaluated after 18 h of IVM. ZEN exposure dose-dependently reduced the proportion of 4-cell/2-cell embryos across all age groups (Figure 6A–C). However, the threshold concentration eliciting statistically significant impairment differed by age: in 3-week-old mice, 5 µM ZEN significantly decreased the 4-cell/2-cell embryo rate relative to vehicle control (Figure 6C); whereas in 6- and 8-week-old mice, this effect only reached statistical significance at 7 µM ZEN (Figure 6B and Figure 6A, respectively).

Similarly, cumulus expansion (quantified as the ratio of AM to BM) remained unaffected by 7 µM ZEN in oocytes from 8-week-old mice (Figure 6D) but was significantly suppressed at 7 µM in 6-week-old mice (Figure 6E) and at 5 µM in 3-week-old mice (Figure 6F). Taken together, these data demonstrate that oocytes from prepubertal mice exhibit intrinsically heightened susceptibility to ZEN-induced functional deficits relative to those from sexually mature adults.

### 3.7. Interaction Between In Vitro ZEN Exposure and Mouse Age on Oocyte Developmental Competence

There was a significant interaction between mouse age and ZEN dose on oocyte activation rate (F = 5.007, *p* = 0.0024 **). The effect of ZEN dose on activation rate varied by mouse age: 3 wk mice showed a 18.1% decrease from 0 to 7 µM, whereas 6 wk and 8 wk mice showed 4.2% and 5.1% reductions, respectively. Blast rate (R^2^ = 0.59), 4C rate (R^2^ = 0.62), and Act rate (R^2^ = 0.66) indicated that age and ZEN dose together explained over 50% of the variation in these parameters.

Except for the 2C rate, all four other developmental indexes were significantly affected by age. Blastocyst rate showed the strongest age effect (F = 15.564, *p* < 0.001 ***), with 8 wk mice having the highest rate (42.6%) and 3 wk mice the lowest (33.8%). In 3 wk mice, 7 µM ZEN reduced blastocyst rate from 39.3% to 30.2% (a relative decline of 23%), whereas in 8 wk mice, the rate dropped only from 45.5% to 40.3% (a 11% reduction). Additionally, Act rate (F = 9.859, *p* < 0.001 ***) and 4C rate (F = 7.662, *p* = 0.002 **) were also significantly influenced by age.

All five developmental indexes were significantly affected by ZEN dose (*p* < 0.05), with Act rate (F = 17.526, *p* < 0.001 ***) and 4C rate (F = 15.515, *p* < 0.001 ***) being the most severely impacted.

In summary, mouse age is an important factor affecting ZEN toxicity: 3 wk mice are most sensitive to ZEN, while 8 wk mice exhibit the highest tolerance. The activation stage is a critical target for ZEN toxicity and demonstrates a significant interaction effect between age and dose.

## 4. Discussion

The present findings demonstrate that oral administration of ZEN to female mice compromises oocyte developmental competence, primarily through induction of oxidative stress and activation of apoptotic pathways. Specifically, ZEN exposure significantly reduced the proportion of 4-cell embryos, impaired cumulus expansion, and dysregulated the expression of key genes associated with oocyte competence and apoptosis resistance, including downregulation of competence-promoting and anti-apoptotic genes. Concurrently, ZEN elevated intracellular ROS levels, diminished MMP, and decreased the ratio of GSH/GSSG, collectively indicating severe redox imbalance. These observations align with prior evidence linking ZEN exposure to oocyte quality deterioration via oxidative damage. For instance, Pan et al. [4] reported that intraperitoneal ZEN injection in female mice induced dose-dependent deficits in oocyte competence, accompanied by oxidative stress, chromatin disorganization, and aberrant transcriptional regulation. Hou et al. [3] observed that dietary ZEN exposure in mice led to marked declines in oocyte quality and mitochondrial dysfunction. Similarly, Lai et al. [13] found that supplementing porcine IVM medium with 3 µM ZEN significantly inhibited polar body extrusion while increasing intracellular ROS and depleting glutathione content.

Moreover, multiple studies have documented ZEN-induced impairment of cumulus expansion across species. Lai et al. [13] demonstrated that in vitro exposure of porcine oocytes to ZEN suppressed both morphological cumulus expansion and mRNA expression of critical expansion-related genes, including HAS2, PTX3, TNFAIP6, and CX43. Wang et al. [14] confirmed that ZEN exposure during porcine IVM significantly disrupted cumulus expansion. In ovine oocytes, ZEN treatment during IVM inhibited cumulus cell proliferation via downregulation of TNFAIP6 [15]. Furthermore, Zhang et al. [16] showed that ZEN injection in female mice markedly reduced the average cumulus expansion area and suppressed Has2 and Ptx3 transcript levels in cumulus cells.

Consistent with the observed reduction in the proportion of 4-cell embryos following ZEN gavage, our RT-qPCR analysis revealed significant downregulation of key genes essential for oocyte developmental competence, including Mater, Zar1, and Prkce. Notably, 2-cell embryonic arrest in NSN mouse oocytes has been linked to the absence of cytoplasmic lattices and concomitant suppression of Mater expression and ribosomal protein synthesis [17]. In Zar1-deficient female mice, fewer than 20% of embryos reach the 2-cell stage, and none progress to the 4-cell stage [18]. Similarly, Prkce knockout mice exhibit a markedly reduced 2-cell rate, a defect fully rescued by microinjection of Prkce cRNA, supporting the utility of Prkce transcript abundance as a predictive biomarker of high-quality mature oocytes [19].

Mechanistically, ZEN might damage oocytes in two possible ways. Firstly, because ZEN is estrogenic, it might disturb production of FSH and LH, which are essential for oocyte development and maturation. It has been reported that ZEN and its derivatives exert an estrogen-like effect and disturb the expression of FSH and LH through estrogen negative feedback [20]. Secondly, ZEN damages oocytes directly, as many studies, including the present one, have shown that in vitro exposure of oocytes to various concentrations of ZEN significantly impaired oocyte competence in various animal species. Furthermore, our use of overdoses of eCG that contain FSH and LH to super-ovulate mice would counteract the estrogen negative feedback effect of ZEN on the expression of FSH and LH.

This study demonstrates that ZEN exposure impairs oocyte developmental competence in an animal age-dependent manner, with prepubertal animals exhibiting the greatest vulnerability. Specifically, oral administration of ZEN at 100 µg/kg to mice aged 3, 6, and 8 weeks resulted in no statistically significant effects on oocyte developmental outcomes in the 8-week-old group; however, the 4-cell:2-cell embryo ratio was significantly diminished in the 6-week-old cohort, while both the 2-cell and 4-cell embryo rates (normalized to activated oocytes) were significantly reduced in the 3-week-old (prepubertal) group. Moreover, although ZEN exposure significantly suppressed the expression of Mater, Zar1, and Prkce, as well as the anti-apoptotic Bcl2/Bax mRNA ratio across all three age groups, the magnitude of downregulation was consistently most pronounced in prepubertal oocytes. While evidence of ZEN’s direct impact on prepubertal oocytes remains limited, epidemiological data strongly implicate ZEN in recurrent outbreaks of hyperestrogenism in livestock, with prepubertal gilts consistently identified as the most susceptible population [7]. Green et al. [10] reported that ZEN exposure in prepubertal gilts significantly lowered mean serum LH concentrations. Furthermore, age-dependent susceptibility is not unique to ZEN: fluoxetine, a selective serotonin reuptake inhibitor, also exerts disproportionately adverse effects on oocyte quality in prepubertal mice compared with older counterparts [21].

However, it remains unclear whether the heightened susceptibility of prepubertal oocytes to ZEN arises from increased ovarian bioavailability of ZEN or from intrinsically greater oocyte sensitivity to its toxic effects. Pharmacokinetic assessment revealed significantly higher ZEN concentrations in both ovarian and hepatic tissues of 3-week-old mice compared with those of 6- and 8-week-old mice, indicating age-dependent accumulation and enhanced intraovarian ZEN exposure during the prepubertal period. Complementing these in vivo findings, IVM experiments, where oocytes isolated from mice of distinct developmental stages were exposed to graded concentrations of ZEN, demonstrated that prepubertal oocytes consistently exhibited greater functional impairment relative to oocytes from sexually mature adults. Collectively, these results indicate that the heightened vulnerability of prepubertal oocytes to ZEN is attributable to a dual mechanism: elevated intraovarian ZEN bioavailability and intrinsically increased oocyte sensitivity.

Accumulation of ZEN and its major metabolites in reproductive tissues, particularly the ovary, has been documented following chronic low-dose exposure in prepubertal gilts [22]. In the present study, systemic ZEN levels in peripheral blood were not quantified, as prior evidence indicates that circulating ZEN concentrations in prepubertal gilts exhibit poor correlation with target-tissue burden, and thus possess limited diagnostic utility [23]. Currently, relatively few studies have directly compared the intrinsic resilience of prepubertal versus adult oocytes to environmental stressors. Paramio et al. [11] reported that prepubertal oocytes display heightened susceptibility to oxidative stress during IVM compared to adult oocytes. Similarly, Jiao et al. [24] demonstrated that prepubertal mouse oocytes exhibit diminished glutathione biosynthesis, resulting in phenotypes consistent with compromised developmental competence such as impaired redox homeostasis, defective male pronucleus formation, and reduced blastocyst yield.

The feed amount mice consume per day is approximately 10% to 15% of their body weight. The equivalent ZEN concentration in feed (µg/kg) = gavage dose (µg/kg BW) ÷ (10–15%). Thus, gavage ZEN doses of 50, 100, and 150 µg/kg BW correspond to ZEN concentrations in feed of approximately 333–500, 667–1000, and 1000–1500 µg/kg, respectively. A large-scale survey in China from 2021 to 2024 revealed average feed ZEN concentrations ranging from 48.0 to 542.2 µg/kg [25]. Among these, the highest was observed in pig feed in 2022, reaching 542.2 µg/kg, with a maximum single sample detection of 3307 µg/kg. Therefore, the equivalent feed concentration for 50 µg/kg BW (333–500 µg/kg) falls within the reported average contamination levels, while that for 150 µg/kg BW (1000–1500 µg/kg feed) exceeds the average but remains below the extreme detected value (3307 µg/kg feed). In summary, the ZEN gavage doses tested in this study fall within the higher end of actual feed contamination levels observed in monitoring data. Furthermore, the present results actually found the NOAEL of ZEN on mouse oocytes following exposure, both in vivo and in vitro.

## 5. Conclusions

This study systematically investigated whether ZEN compromises oocyte developmental competence in an animal age-dependent manner and found that prepubertal oocytes are significantly more susceptible to ZEN-induced developmental failure than adult oocytes. Further observations indicate that this vulnerability is associated synergistically with (i) elevated intraovarian ZEN accumulation and (ii) intrinsically greater sensitivity to ZEN of the oocyte itself. These findings advance the mechanistic understanding of mycotoxin-mediated reproductive toxicity and underscore the critical need for stringent ZEN contamination control—not only to safeguard normal pubertal development in livestock but also to ensure the efficacy and safety of juvenile in vitro embryo transfer (JIVET).

## Figures and Tables

**Table 1:** Sequences of primers used for one-step RT-PCR in this study.

Genes	Forward Primer (5′-3′)	Reverse Primer (5′-3′)
*Mater*	TCAAGAACTGGAACTAGTGGAC	GCTTGGTTGTTGTGATCATACA
*Zar1*	GAGCAGAAGTACGGCTACTATC	CACTCGGCAGAACTGTTTGAA
*Prkce*	CAGAATGGGAGCCGTCACTTC	CCTGAACACTCGTTCTTCATTGT
*Bcl2*	GAGCGTCAACAGGGAGATG	GGGCCATATAGTTCCACAAAGG
*Bax*	TGCAGAGGATGATTGCTGAC	GATCAGCTCGGGCACTTTAG
*H2afz*	ACAGCGCAGCCATCCTGGAGTA	TTCCCGATCAGCGATTTGTGGA

**Table 2:** Effects of Z100 gavage on the ZEN contents (µg/kg tissue) in ovaries and livers of mice at different ages.

Age (Weeks)	Mice Used	Ovaries	Livers
8	36	0.09 ± 0.01 ^a^	0.07 ± 0.01 ^a^
6	36	0.12 ± 0.01 ^a^	0.09 ± 0.01 ^a^
3	36	0.17 ± 0.02 ^b^	0.17 ± 0.01 ^b^

^a^, ^b^: Values with a different letter in superscripts differ significantly (*p* < 0.05). Each treatment was repeated 3 times, with each replicate containing tissue samples from 12 mice.

## Data Availability

Data will be made available on request.

## Supplementary Materials

The following supporting information can be downloaded at: https://www.mdpi.com/article/10.3390/cells15161444/s1, Figure S1: Calibration curve for ZEN; Figure S2: Residual plot for ZEN calibration curve; Figure S3: Representative MRM chromatograms of ZEN in tissue homogenates.

## Abbreviations

The following abbreviations are used in this manuscript:

AM	After maturation
BM	Before maturation
CB	Cytochalasin B
COCs	Cumulus–oocyte complexes
DCHF-DA	2′,7′-dichlorodihydro-fluorescein diacetate
DMSO	Dimethylsulfoxide
DOs	Cumulus-denuded oocytes
eCG	Equine chorionic gonadotropin
EGF	Epidermal growth factor
FSH	Follicle stimulating hormone
GSH	Reduced glutathione
GSSG	Oxidized glutathione
GSX	Total glutathione
GV	Germinal vesicle
LC–MS/MS	Liquid chromatography–tandem mass spectrometry
LH	Luteinizing hormone
IVM	In vitro maturation
MII	Metaphase II
MMP	Mitochondrial membrane potential
ROS	Reactive oxygen species
ZEN	Zearalenone
ZEN14G	Zearalenone-14-glucoside
ZEN14S	Zearalenone-14-sulfate
