# Supplementary material for: Animal Age-Dependent Susceptibility of Mouse Oocytes to Zearalenone-Induced Developmental Impairment: Roles of Pharmacokinetic Exposure and Intrinsic Oocyte Sensitivity
Source: Cells. 2026 Aug 11;15(16):1444. doi: 10.3390/cells15161444 (PMC13511373; doi:10.3390/cells15161444)
Supplement: Supplementary file 1 [file cells-15-01444-s001.zip › cells-4441567-supplementary.pdf]

Si-Tong Liu et al. Supplementary figures

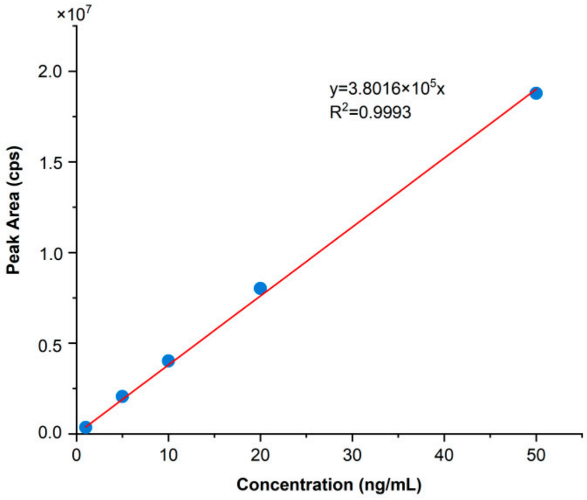

Figure S1. Calibration curve for ZEN. Blue dots represent calibration standards in the range of 1.0-50.0 ng/mL, and the red solid line represents the linear regression fit (R^2=0.9993). The linear regression equation is shown in the figure. The back-calculated accuracy of all calibration standards ranged from 91.9% to 108.1%, meeting the acceptance criteria of ±15% for bioanalytical method validation.

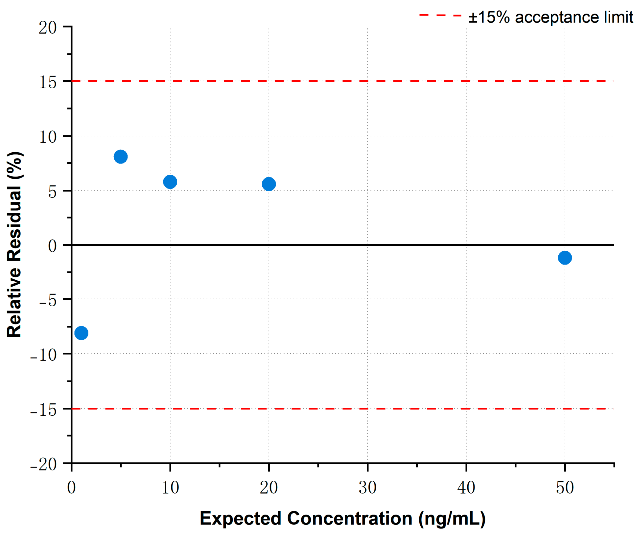

Figure S2. Residual plot for ZEN calibration curve. The x-axis represents the expected concentrations of the calibration standards (ng/mL), and the y-axis represents the relative residual (%) for each calibration point. The red dashed lines indicate the ±15% acceptance limits commonly applied in bioanalytical method validation. All relative residuals fell strictly within the ±15% acceptance range (distribution from -8.1% to +8.1%), with no obvious systematic bias, indicating that the linear regression model provided a satisfactory fit.

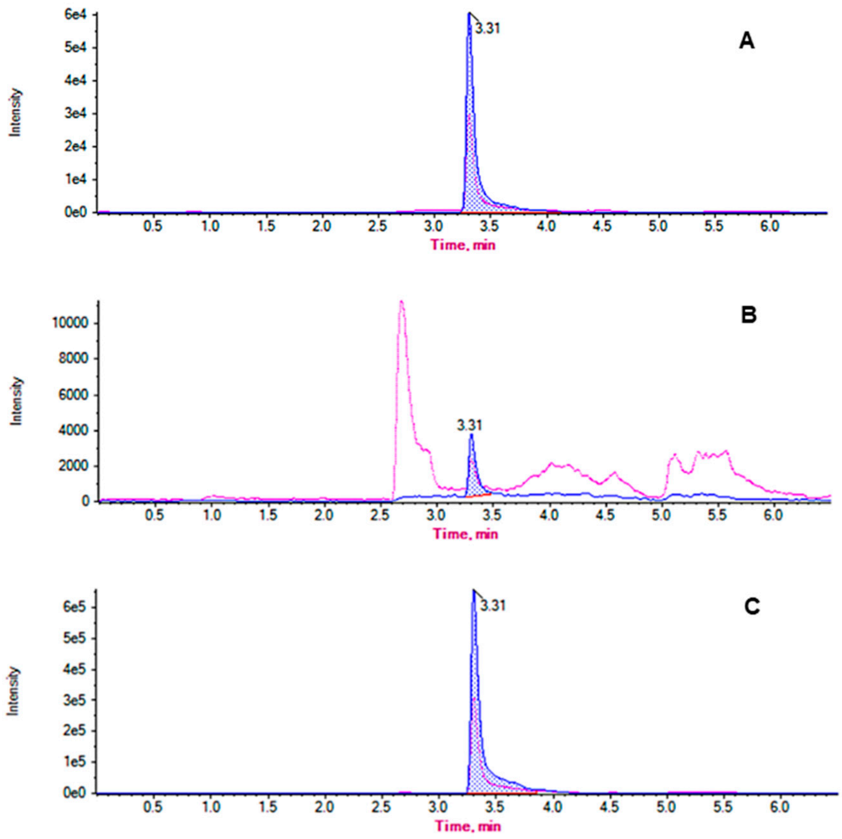

Figure S3. Representative MRM chromatograms of ZEN in tissue homogenates. (A) ZEN standard at 1 ng/mL, retention time (RT) = 3.31 min, peak area = 3.49×10^5 cps; (B) Blank liver homogenate (un-spiked matrix control). The background signal at the ZEN retention time (3.31 min) was 4.14×10^3 cps, corresponding to only 1.2% of the peak area of the 1 ng/mL standard, confirming the absence of significant endogenous matrix interference. Additionally, matrix-derived components eluting at 2.6 min and 3.8-5.5 min were well separated from the ZEN peak, further ensuring method specificity; (C) Liver homogenate spiked with ZEN at 10 ng/mL, RT = 3.31 min, peak area = 3.998×10^6 cps, with a spike recovery of 105%, falling within the acceptable range (85%-115%). Note that the y-axis scales differ among panels.
